# Development of a quadruplex loop-mediated isothermal amplification assay for field detection of four *Vibrio* species associated with fish disease

**DOI:** 10.1186/s40064-016-2760-x

**Published:** 2016-07-16

**Authors:** Shun Zhou, Zhi-xin Gao, Min Zhang, Dan-yang Liu, Xin-peng Zhao, Yong Liu

**Affiliations:** Marine Science and Engineering College, Qingdao Agricultural University, Qingdao, 266109 People’s Republic of China; College of Fisheries, Qingdao Ocean University, Qingdao, 266100 People’s Republic of China

**Keywords:** A quadruplex LAMP method, *Vibrio ichthyoenteri*, *Vibrio parahaemolyticu*s, *Vibrio scophthalmi*, *Vibrio vulnificus*, Field detection

## Abstract

A quadruplex loop-mediated isothermal amplification (LAMP) method was developed to detect four *Vibrio* species, including *Vibrio ichthyoenteri*, *Vibrioparahaemolyticu*s, *Vibrioscophthalmi*, and *Vibrio vulnificus*, simultaneously. Four sets of species-specific primers were designed with different restriction sites contained in the inner primers. The quadruplex LAMP method could distinguish four *Vibrio* species via the subsequent restriction enzyme analysis. The sensitivity of the quadruplex LAMP method were 10^2^–10^3^ times higher than the sensitivity of conventional PCR. *V. scophthalmi*, *V. vulnificus*, *V. parahaemolyticu*s and *V. ichthyoenteri* could be detected in the different tissues of the infected fish by the quadruplex LAMP method simply and conveniently through using SYBR Green I to facilitate visual inspection of the LAMP products. The method we developed in this study could be a simple and convenient diagnostic tool for field detection of *Vibrio* infection in fish.

## Background

In aquaculture, vibriosis is known as a major bacterial disease in fish culture systems and can cause considerable loss in terms of production and processing (Toranzo et al. 2005). Many *Vibrio* species have been recognized as fish pathogens that can cause infection with various symptoms. For example, *Vibrio scophthalmi* infection results in hemorrhage on fish body surface and inner surface of the abdomen, severe enteritis, and ascites (Qiao et al. 2012); *Vibrio ichthyoenteri* infection cause opaque intestines and necrotizing fasciitis with high mortality rates (Ishimaru et al. 1996; Lee et al. 2012); *Vibrioparahaemolyticus* infection causes diseases not only in fish, shrimp, oysters, and mussels, etc. (Montilla et al. 1994; Quintoil et al. 2007), but also is important in public health and causes gastrointestinal disorders in humans who ingest contaminated fish and shellfish (Kubota et al. 2008; Iwamoto et al. 2010); and *Vibriovulnificus* has been associated with vibriosis outbreaks in fish and shellfish (Haenen et al. 2014) and can also cause severe, progressive necrotizing infection in human (Strom and Paranjpye 2000).

Rapid identification of the vibriosis pathogens to the species level is useful for research and epidemiological studies because identification can help with determining the exact source of any outbreak and in developing strategies to reduce the severity of the disease. However, the traditional identification techniques, which consist of a series of isolations on selective agar medium followed by biochemical and serological testing, are time-consuming and ambiguous (Harwood et al. 2004; Akond, et al. 2008). An array of molecular techniques has been gaining popularity for the identification of different aquaculture-related bacterial pathogens and includes the following: PCR-based identification methods for targeting 16S–23S rRNA intergenic spacer regions among vibrio species, including *V. parahaemolyticus, V. vulnificus*, etc. (Maria et al. 2010), a multipex PCR method was developed by using the *rpo*B gene to make the identificationg of *Vibrio harveyi*, *V. ichthyoenteri*, and *Photobacterium damselae* (Myoung et al. 2014), colony hybridization by species-specific probes to identify *V. scophthalmi* in the intestinal microbiota of fish and an evaluation of host specificity (Cerdà-Cuéllar and Blanch 2002), multiprobe fluorescence in in situ hybridization for the rapid enumeration of viable *V. parahaemolyticus* (Sawabe et al. 2009), and a simple and rapid PCR-fingerprinting method for *V. cholerae* on the basis of genetic diversity of the superintegron (Chowdhury et al. 2010).

The loop-mediated isothermal amplification (LAMP) method, developed in 2000 by Notomi et al. (2000) as a novel nucleic acid detection, is a desirable diagnostic tool for on-site epidemiological investigations of bacterial infection. Relying on its convenient operation, the short time required for results, and the high specificity, the LAMP method has been used in aquaculture as an effective method for pathogen detection. LAMP has been used widely for *Vibrio* detection in fish disease and shows high specificity, sensitivity and rapidity under isothermal conditions when used to identify a single *Vibrio.* Single pathogen LAMPs have been developed for *Vibrio parahaemolyticus* (Yamazaki et al. 2008), *Vibrio nigripulchritudo* (Fall et al. 2008), and *Vibrio alginolyticus* (Cai et al. 2010). In addition, many multiplex loop-mediated isothermal amplification (mLAMP) methods emerged in response to the need to detect two or more pathogens in one reaction system, and these mLAMP assays combined the LAMP technique with restriction enzyme analysis, constructing an original cleavage site within the amplification products rather than within the designed primers (Iwamoto et al. 2003; Iseki et al. 2007) to make this method more convenient and efficient in practice. This method has been used to confirm whether the amplification products are rooted in the target genes. He and Xu (2011) have successfully reported an mLAMP that detected two virus-inserted restriction enzyme cleavage sites in two pairs of species-specific primers, and Yu et al. (2013) developed a triLAMP (triplex loop-mediated isothermal amplification, triLAMP) method for detecting three *Vibrio* species successfully by designing primer sets with one or two restriction enzyme sites contained in the inner primers of each set. In addition, the results can be detected with the naked eyes by the addition of SYBR Green I, which is an important advantage in the development of a simple and rapid diagnostic tool.

In the present study, we developed an assay based on the LAMP technique for simultaneous detection of four *Vibrio* species in fish, and investigated its sensitivity, specificity, and application potential in fish. Our results indicated that the quadruplex LAMP method we constructed could identify four *Vibrio* species rapidly and accurately. This method will greatly help to detect pathogenic bacteria in fish farms.

## Results and discussion

### Optimization of the LAMP reaction conditions

**Fig. 1:**
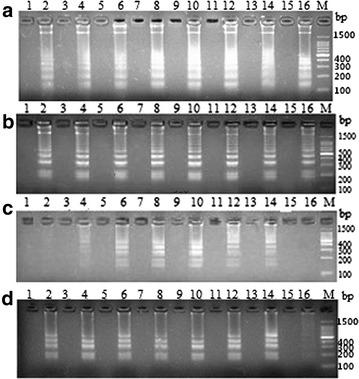
The optimization of the LAMP reaction temperature. LAMP reaction temperature of *V. scophthalmi* (**a**), *V. vulnificus* (**b**), *V. parahaemolyticus* (**c**) and *V. ichthyoenteri* (**d**) was set at 58–65 °C with 1 °C intervals, respectively. *Lanes* *2*, *4*, *6*, *8*, *10*, *12*, *14*, and *16* were the amplification products*, lanes 1, 3, 5, 7, 9, 11, 13*, and *15* were negative control (the application templates used was ddH_2_O). *M* Marker. All products were electrophoresed on a 2 % agarose gel

To determine the optimal reaction temperature and time, the uni-LAMP was conducted using DNA template of *V. scophthalmi, V. vulnificus, V. parahaemolyticu*s and *V. ichthyoenteri*. The results of the LAMP amplification products by gel electrophoresis indicated that *V. scophthalmi* was detected at 58, 59, 60, 61, 62, 63, 64, and 65 °C (Fig. 1a); *V. vulnificus* was detected at 58, 59, 60, 61, 62, 63, 64, and 65 °C (Fig. 1b); *V. parahaemolyticu*s was detected at 60, 61, 62, 63, and 64 °C (Fig. 1c); and *V. ichthyoenteri* was detected at 58, 59, 60, 61, 62, 63, and 64 °C (Fig. 1d). The 62 °C temperature was chosen for the subsequent assays on the basis of the brightness of the electrophoretic bands.

**Fig. 2:**
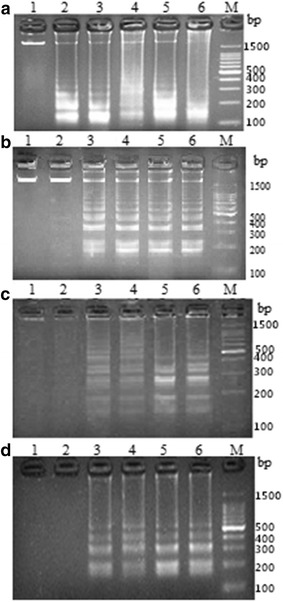
The optimization of LAMP reaction time. LAMP reaction time for *V. scophthalmi* (**a**), *V. vulnificus* (**b**), *V. parahaemolyticus* (**c**) and *V. ichthyoenteri* (**d**) was set at 15–90 min with 15 min intervals corresponding to *lanes 1, 2, 3, 4, 5*, and *6*. *M* Marker. All products were electrophoresed on a 2 % agarose gel

At 62 °C, the LAMP products with the *V. scophthalmi* template displayed clear bands when the reaction was performed for 30, 45, 60, 75 and 90 min (Fig. 2a), whereas the LAMP products with the *V. vulnificus, V. parahaemolyticu*s and *V. ichthyoenteri* template displayed clear bands when the reaction was performed for 45, 60, 75 and 90 min (Fig. 2). The 75 min reaction time was chosen as the time in which the subsequent assays were conducted based on the brightness of the electrophoretic bands. Based on these results, the optimal quadruplex LAMP reaction conditions were 62 °C for 75 min.

### Specificities of the quadruplex LAMP method

To examine the specificity of the quadruplex LAMP method, the assays were carried out with DNA templates of bacterial strains that included 26 *Vibrio* strains and 5 non-*Vibrio* strains (Table 1). The results showed positive results for all tested target *Vibrio* species (Table 1), whereas the other strains showed negative results (Table 1).

**Table 1:** Bacterial strains used in this study

Strains	Sources	The amplification results
*Escherichia coli* DH5α	Transgen Biotech	–
*Micrococcus luteus* 1D00051	MCCC	–
*Pseudomonas putida* C1	Preserved in laboratory	–
*Staphylococcus aureus* 1D00101	MCCC	–
*Streptococcus agalactiae* G1	Preserved in laboratory	–
*Vibrio anguillarum* CJ	Preserved in laboratory	–
*Vibrio harveyi* Z1	Preserved in laboratory	–
*Vibrio ichthyoenteri* 1A00057	MCCC	+
*Vibrio ichthyoenteri* 1A00059	MCCC	+
*Vibrio ichthyoenteri* 1B00564	MCCC	+
*Vibrio ichthyoenteri* 1B00627	MCCC	+
*Vibrio ichthyoenteri* 1B00641	MCCC	+
*Vibrio ichthyoenteri* 1B00689	MCCC	+
*Vibrio ichthyoenteri* 1B01039	MCCC	+
*Vibrio ichthyoenteri* 1B00	MCCC	+
*Vibrio parahaemolyticu*s PL2	Preserved in laboratory	+
*Vibrio* *parahaemolyticu*s Yh1	Preserved in laboratory	+
*Vibrio* *parahaemolyticu*s 17802	ATCC	+
*Vibrio* *parahaemolyticu*s 1A02609	MCCC	+
*Vibrio* *parahaemolyticu*s 1H00015	MCCC	+
*Vibrio* *parahaemolyticu*s 1A10122	MCCC	+
*Vibrio* *parahaemolyticu*s 1K02691	MCCC	+
*Vibrio* *parahaemolyticu*s 1A02609	MCCC	+
*Vibrio* *vulnificus* PR1	Preserved in laboratory	+
*Vibrio* *vulnificus* ZS1	Preserved in laboratory	+
*Vibrio* *vulnificus* 27562	ATCC	+
*Vibrio* *vulnificus* 1H00066	MCCC	+
*Vibrio* *vulnificus* 1A08743	MCCC	+
*Vibrio* *vulnificus* 1H00047	MCCC	+
*Vibrio* *vulnificus* 1B00281	MCCC	+
*Vibrio scophthalmi* ZS1	Preserved in laboratory	+

With respect to the specificity level achieved in this study, the specific species could be discriminated by restriction enzyme analysis because different types of restriction enzyme cutting sites were introduced to the primers targeting these four *Vibrio* species. In addition, the assays indicated that only the tested strains belonging to *V. scophthalmi*, *V. vulnificus*, *V. parahaemolyticu*s and *V. ichthyoenteri* showed positive results, whereas other strains representing two other *Vibrio* species and five non-*Vibrio* species showed negative results.

### Differential identification of the *Vibrio* species in the quadruplex LAMP

**Fig. 3:**
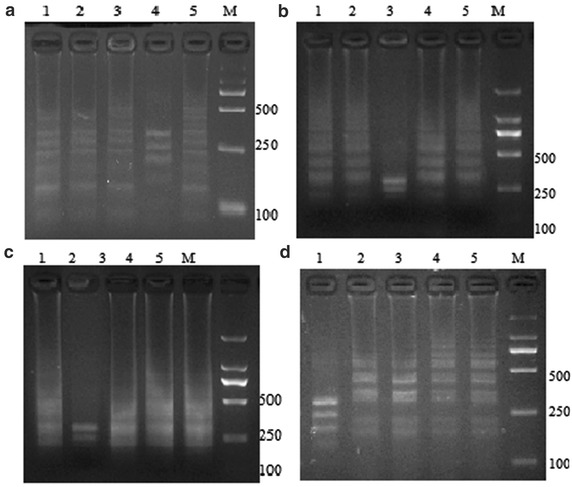
Construction of the quadruplex LAMP reaction. The quadruplex LAMP products (**a** *V. scophthalmi*, **b** *V. vulnificus*, **c** *V. parahaemolyticus*, **d** *V. ichthyoenteri*) and their restriction enzyme-digestion maps. *Lane 1* *EcoR*V digested products, *Lane 2* *Pst* I digested products, *Lane 3* *BamH* I digested products, *Lane 4* *EcoR* I digested products, *Lane 5* amplification products. All the quadruplex LAMP-amplified products and digested products were subjected to electrophoresis on a 2 % agarose gel

To identify the specific bacterial species which causes positive quadruplex LAMP result, the LAMP products were subjected to restriction enzyme digest. The results showed that the quadruplex LAMP products of *V. scophthalmi* could be only digested by *EcoR*I, while those of *V. vulnificus*, *V. parahaemolyticu*s and *V. ichthyoenteri* could only be digested by *BamH*I, *Pst* I and *EcoR*V, respectively. The digests yielded a few small-size bands, which were distinguishable from those of LAMP products (Fig. 3). Hence, following the quadruplex LAMP reaction, the bacterial species can be easily identified by simple treatments of the amplicons with four different restriction enzyme digest systems.

### Sensitivity of the quadruplex LAMP method

**Fig. 4:**
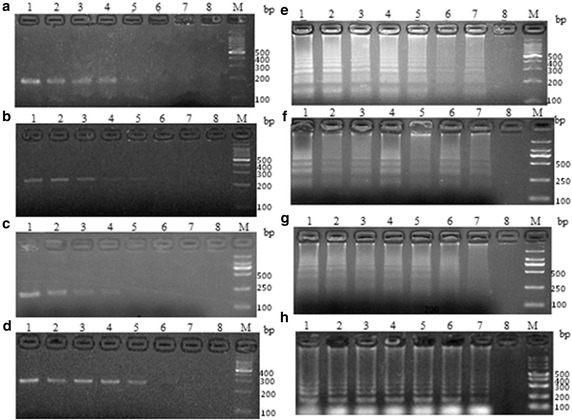
Sensitivities of the quadruplex LAMP and conventional PCR methods. *Lanes 1–9* the quadruplex LAMP (**e**–**h**) and general PCR (**a**–**d**) were carried out using DNA extracted from different concentrations of *V. scophthalmi* (**a**, **e**), *V. vulnificus* (**b**, **f**), *V. parahaemolyticus* (**c**, **g**) and *V. ichthyoenteri* (**d**, **h**) as template. The bacteria concentration from *lanes 1–8* were 8 × 10^6^, 8 × 10^5^, 8 × 10^4^, 8 × 10^3^, 8 × 10^2^, 8 × 10^1^, 8 × 10^0^, and 8 × 10^−1^ CFU per reaction, respectively. *M* Marker. All products were electrophoresed on a 2 % agarose gel

Compared to conventional PCR methods, the detection limits of the quadruplex LAMP method rely on the initial inocula of *V. scophthalmi*, *V. vulnificus*, *V. parahaemolyticu*s and *V. ichthyoenteri* (8 × 10^8^ CFU ml^−1^). A tenfold serial dilution of the culture was used, and the corresponding DNA was used for the subsequent quadruplex LAMP reaction and PCR. The LAMP reaction was able to detect *V. scophthalmi*, *V. vulnificus*, *V. parahaemolyticu*s and *V. ichthyoenteri* up to 8 × 10^3^ CFU ml^−1^ (8 CFU per reaction) (Fig. 4), whereas the conventional PCR could detect *V. scophthalmi*, *V. vulnificus*, *V. parahaemolyticu*s and *V. ichthyoenteri* up to 8 × 10^5^ CFU ml^−1^ (8 × 10^2^ CFU per reaction) (Fig. 4a), 8 × 10^5^ CFU ml^−1^ (8 × 10^2^ CFU per reaction) (Fig. 4b), 8 × 10^6^ CFU ml^−1^ (8 × 10^3^ CFU per reaction) (Fig. 4c) and 8 × 10^5^ CFU ml^−1^ (8 × 10^2^ CFU per reaction) (Fig. 4d), respectively. The sensitivities of the quadruplex LAMP method were 10^3^ times higher than observed for the PCR detection of *V. parahaemolyticu*s and 10^2^ times higher than conventional PCR in detecting *V. scophthalmi*, *V. vulnificus* and *V. ichthyoenteri.*

Some differences exist between our method and methods previously reported for LAMP-based detection. In this study, we followed the protocol modified by Yu et al., who utilized crude tissue homogenates instead of the extracted DNA as templates and avoided having to use laboratory instruments (such as a centrifuge) that required for the DNA extraction process. The sensitivity of the quadruplex LAMP assay that positively detected among the *V. scophthalmi*, *V. vulnificus*, *V. parahaemolyticu*s and *V. ichthyoenteri* was 10^2^–10^3^ times higher than the sensitivity of the conventional PCR and was similar to the monoplex LAMP method and triplex reported previously (Mao et al. 2012; Yu et al. 2013).

### Applicability of the quadruplex LAMP method for detecting Vibrio-infected fish under field conditions

**Fig. 5:**
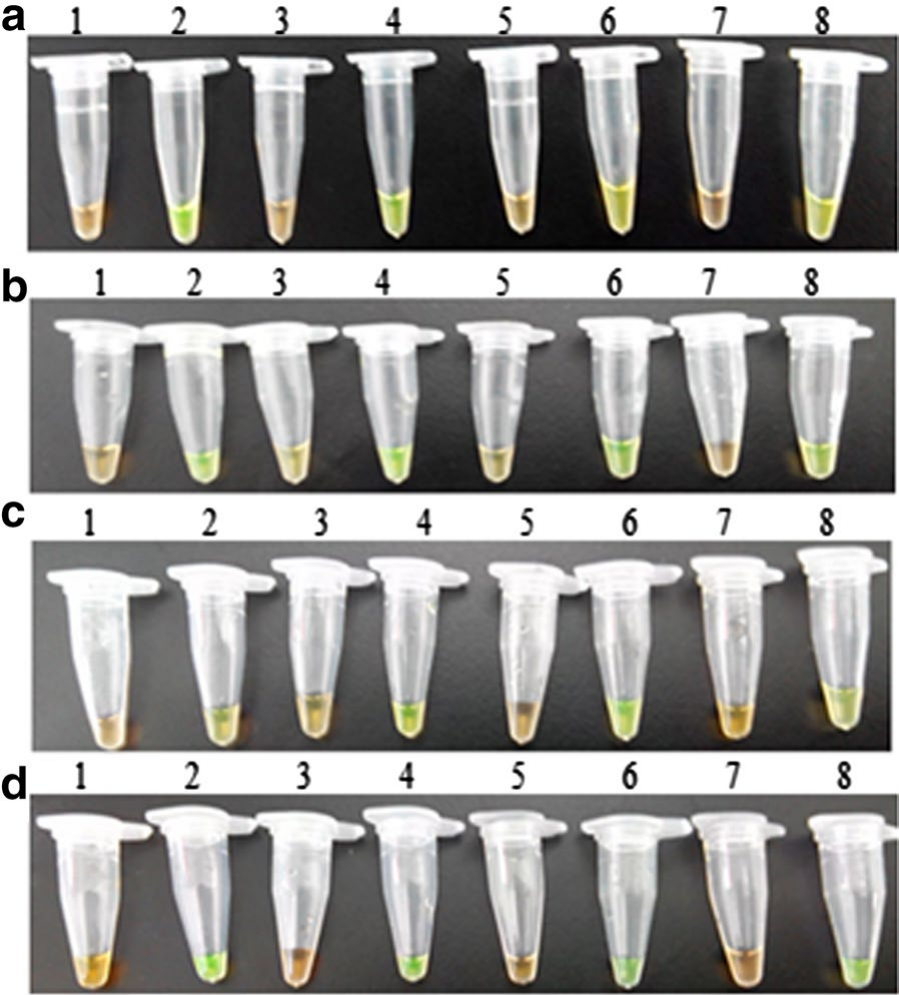
Visual inspection of the quadruplex LAMP results. The boiled homogenates of the tissues collected from the fish that had been experimentally injected with PBS or vibrios (**a** *V. scophthalmi*, **b** *V. vulnificus*, **c** *V. parahaemolyticus*, and **d** *V. ichthyoenteri*) were used as templates. In all panels, *Lane 1, 3, 5*, and *7* represents spleen, kidney, liver and blood from the PBS-injected fish, respectively; *Lane 2, 4, 6*, and *8* represents spleen, kidney, liver, and blood from the infected fish, respectively

To determine the practical applications of the quadruplex method for detecting *Vibrio* in fish, we utilized the boiled homogenates of various tissues such as blood, kidney, spleen and liver isolated from turbot, *Scophthalmus maximus* experimentally infected with *V. scophthalmi*, *V. vulnificus*, *V. parahaemolyticu*s and *V. ichthyoenteri.* The assays were conducted with only heating equipment and a water bath pot. Both are available on fish farms. The results indicated that four *Vibrio* species could be detected in all tissue samples by visual judgments of the quadruplex LAMP products stained with the SYBR Green I (Fig. 5), which is an important advantage in the development of a simple and rapid diagnostic tool.

**Fig. 6:**
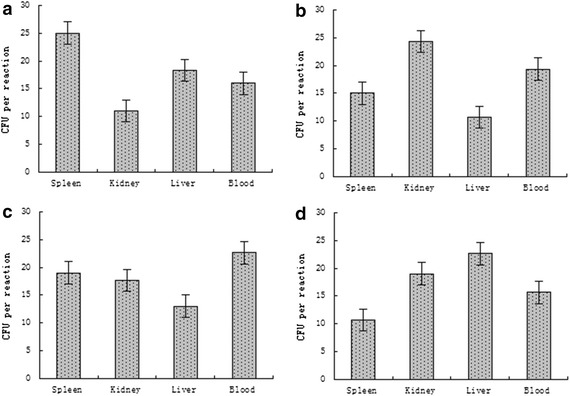
The detection limits of the quadruplex LAMP method under site conditions. Blood, kidney, liver and spleen were collected from *S. maximus* infected with *V. scophthalmi* (**a**), *V. vulnificus* (**b**), *V. parahaemolyticus* (**c**), and *V. ichthyoenteri* (**d**), respectively. Tissue homogenates and blood were used as templates in the quadruplex LAMP method, which was conducted under field conditions. The detection limits, which are represented by bacterial numbers (of each reaction), are indicated on the *Y-axis*. Data are shown as the mean ± SD (N = 3)

To evaluate the sensitivity of the quadruplex LAMP method in the blood, kidney, spleen and liver of infected fish by electrophoresis, the detection limits were 16 CFU per reaction, 11 CFU per reaction, 25 CFU per reaction, and 18 CFU per reaction, respectively, for *V. scophthalmi;* 19 CFU per reaction, 24 CFU per reaction, 15 CFU per reaction, and 10 CFU per reaction, respectively, for *V. vulnificus;* 23 CFU per reaction, 17 CFU per reaction, 19 CFU per reaction, and 13 CFU per reaction, respectively, for *V. parahaemolyticu*s; and 15 CFU per reaction, 20 CFU per reaction, 10 CFU per reaction, and 22 CFU per reaction, respectively, for *V. ichthyoenteri* (Fig. 6). These results demonstrate that the quadruplex LAMP method was a feasible pathogenic diagnostic procedure for sensitive on-site detection of *V. scophthalmi*, *V. vulnificus*, *V. parahaemolyticu*s and *V. ichthyoenteri.*

To compare the sensitivity between electrophoresis and visual inspection (SYBR Green I), SYBR Green I was added into the tube at the end of the quadruplex LAMP, the results showed the detection limits were similar with that of electrophoresis (data not shown).

The limits of the quadruplex LAMP method for detecting *Vibrio* in fish tested using boiled homogenates of blood, kidney, liver and spleen from *S. maximus* infected with *V. scophthalmi*, *V. vulnificus*, *V. parahaemolyticu*s and *V. ichthyoenteri* ranged from 10 CFU per reaction to 25 CFU per reaction in the practical applicability. These limits prove that this method could satisfy the need for early diagnosis of *Vibrio* infection in fish and has the potential to be applied in aquaculture to avoid the tedious DNA extraction process. Furthermore, Yu et al. (2013) reported that the sensitivity of an on-site triLAMP method that utilized crude tissue homogenates instead of extracted DNA was similar to the triLAMP method with DNA template. Our findings showed that the detection limit results described from the use of crude tissue homogenates are similar to the quadruplex LAMP method with DNA template. These results were equal to the triplex loop-mediated isothermal amplification method reported previously (Yu et al. 2013).

## Conclusions

In this study, we developed a quadruplex LAMP assay to achieve rapid, efficient and convenient detection of four *Vibrio* species, including *V. scophthalmi*, *V. vulnificus*, *V. parahaemolyticu*s and *V. ichthyoenteri.* This method is suitable for use under field conditions, and is helpful for fisherman to take emergency measures in order to prevent the spread of infection.

## Methods

### Bacterial species

The bacterial strains used in this study were listed in Table 1. Except for *Staphylococcus aureus*, which was cultured in Brain Heart Infusion (BHI) broth, all other strains were cultured in Luria–Bertani (LB) medium. All strains were cultured at 37 °C (for *Escherichia coli*, *Micrococcus luteus* and *Staphylococcus aureus*) or 28 °C (for all others).

### DNA extraction

Bacteria cultured overnight(about 12 h) were washed three times with cold phosphate-buffered saline (PBS) by centrifuging at 11,000×*g* for 5 min and then resuspended in PBS and stored at 4 °C. The tenfold serial dilution plate counting method was used to determined the number of bacteria in a given population: at first, obtain 8 small, sterile test tubes, label the tubes 1 through 8 and then add 4.5 ml of PBS to each test tube, pipette 0.5 ml of the original bacterial cultured into test tube 1, and mixed thoroughly (using the vortexers on each bench) before proceeding to the next step, then obtained a clean pipette and withdraw 0.5 ml of the diluted bacterial suspension from the first test tube and pipette that into the second test tube. Continued in this fashion until we serially diluted the original bacterial suspension into test tube 8, the dilutions from test tube 1 to test tube 8 were from 1/10–1/10^8^, the next step, just pipetted 0.5 ml of the diluted suspension from the appropriately diluted test tube onto the surface of the LB plate, after 18 h, calculated the number of colony forming units (CFU) on your plates. To calculate the number of bacteria per ml of diluted sample one should use the following equation:

$${\text{Number}}\;{\text{of}}\;{\text{CFU}}/\left( {{\text{Volume}}\;{\text{plated}}\;\left( {\text{mL}} \right) \times {\text{total dilution used}}} \right) = {\text{Number of CFU}}/{\text{mL}}$$

The concentration of the bacteria was determined by the serial dilution plate counting method mentioned above and adjusted to 8 × 10^9^ CFU ml^−1^. The suspensions were boiled for 10 min and then centrifuged at 11,000×*g* for 5 min at 4 °C. The supernatants were transferred into new tubes and used immediately for LAMP reaction.

### LAMP primers design

Based on the sequences of the *lux*R gene of *V. scophthalmi* (GenBank accession no. JN684209.1), the metalloprotease gene of *V. vulnificus* (GenBank accession no. U50548.1), the *omp*A gene of *V. parahaemolyticu*s (GenBank accession no. JTGT01000603.1), and the *Tox*R gene of *V. ichthyoenteri* (GenBank accession no. KT265743), four sets of LAMP primers were designed with Primer Explorer 4.0 online software according to the principles proposed by Notomi et al. (2000) (Table 2). Each set contained four primers matching a total of six distinct fragments (from inner to outer: F1/F1c, F2/F2c, F3/F3c and B1/B1c, B2/B2c, B3/B3c) of the target gene, i.e., FIP against F1 and F2c, F3 primer against F3, BIP against B1 and B2c and B3 primer against B3. The inner primers were modified by inserting different restriction enzyme cleavage sites to the linking regions, in order to distinguish among different *Vibrio* species. For the primers designed for detecting *V. scophthalmi*, *V. ichthyoenteri*, *V. vulnificus* and *V. parahaemolyticu*s, a *Eco*RI, *Eco*RV, *Bam* HI or *Pst* I site was introduced to FIP between F1c and F2, or BIP between B1c and B2 as shown in Table 2.

**Table 2:** The primers used in this study

Primers	Type	Sequence (5′^–^3′)	Length (bp)
*lux*R-F3	F3	AGCAAAAAGACCCCGCAC	18
*lux*R-B3	B3	CGGTTTCGTTCTCGGTGTT	20
*lux*R-FIP	F1c F2	GGCGCGCAAATACATCCAGAGAA-GAATTC- GACTCTCGCCCAAAAAACGT (*EcoR*I)	49
*lux*R-BIP	B1c B2	GTGGGATTGGTCGTGGTGGT-TTTT- ATACCGTGGCGACCGATAC	43
metalloprotease-F3	F3	TCAGCAAACCTATTTGGGCC	20
metalloprotease-B3	B3	GTCTTCACGTGTTGGGAAGT	19
metalloprotease-FIP	F1c F2	CCATGCACATTGCTCAACCCTG-TTTT- CCTGTGTTTGATACCGCCG	45
metalloprotease-BIP	B1c B2	GATCAGCAACAAGCCATCGCC-*GGATCC*- TACGATTGGCATGCTTCGC (*Bam*HI)	46
*omp*A-F3	F3	AATTACGCGGAAAGAAACC	19
*omp*A-B3	B3	GAGGCTTATTTCAATAACATGC	22
*omp*A-FIP	F1c F2	AGCGGTAGGTTACTCATCCTCTAA-TTTT- GGATCGCTTCTCATCTTCA	47
*omp*A-BIP	B1c B2	TTACCCACCCGTAGTGTTCG-*CTGCAG*- CAATCGAGAACTCGTGCC (*Pst*I)	44
*Tox*R-F3	F3	TTTGCCGCTCAGCTAACC	18
*Tox*R-B3	B3	CCCAACCCCAAGTTGAGTT	19
*To*xR-FIP	F1c F2	AAGCGTAACCATGCCGCCAC-GATATC- TACGCGTGGTGTCTATAGCA (*Eco*RV)	46
*Tox*R-BIP	B1c B2	AGTTCAGCCATGAAGTTGGGCA-TTTT- TGGTTTATGAATTGCCCCCT	46

### LAMP reaction

The quadruplex LAMP was carried out in a 25 μl reaction volume containing 0.5 μl (1.6 μM) each of the two inner primers (FIP and BIP), 0.5 μl (0.4 μM) each of the two outer primers (F3, B3), 2.5 μl dNTPs (2.5 mM), 0.8 M betaine, and 1 μl (8 U) BstDNA polymerase with its corresponding 10× ThermoPol Buffer, and 1 μl DNA template. The mixture was incubated in a conventional heat block at 58–65 °C with 1 °C intervals for 60 min, and subsequently at 80 °C for 5 min for the termination. The method on electrophoresis combined with five parts: first step, prepare 1× TBE solutions and poured 2 % agarose gel, second step, add 1/6 volume of 6× loading buffer (1 μl) to amplification products (5 μl) and mixed well, the third step-electrophoresis, voltage 100 V, electrical current 60 mA, time 30 min, the fourth step, remove the gel and visualize bands of DNA under UV (Ultraviolet) light, take pictures. The amplification products were electrophoresed on 2 % agarose gel to confirm the optimal reaction temperature at which the amplification products of all four *Vibrio* species showed clear ladder banding. The reaction time (15, 30, 45, 60, 75 and 90 min) was optimized, based on the same principle used in the temperature optimization. To prove the primers were species-specific among the target *Vibrio* species, uni-LAMP assays were carried out under the optimization conditions with the DNA extracted from *V. scophthalmi, V. vulnificus, V. parahaemolyticu*s and *V. ichthyoenteri.*

The quadruplex LAMP assay was implemented under the optimal conditions determined above, and the procedure was identical to that of the uni-LAMP, except that four sets of primers (Table 2) were substituted for the one primer set used in the uni-LAMP. To determine the specificity of the quadruplex LAMP method, the quadruplex LAMP was carried out with the DNA template of 31 bacteria strains, whereas the sensitivity of the method was estimated using the DNA from a tenfold serial dilution of each initial adjusted concentration of bacteria (8 × 10^9^ CFU ml^−1^).

### Restriction enzyme-digestion of the amplified DNA products

To identify bacteria species (*V. scophthalmi, V. vulnificus, V. parahaemolyticu*s and *V. ichthyoenteri*) corresponding to application products, restriction enzyme-digestion of the products were carried out. Each 5 μl of reaction products were digested with the *Eco*RI, *Bam*HI, *Pst* I and *Eco*RV, respectively, and incubated at 37 °C for 1 h. The quadruplex LAMP products and digested DNA products were subjected to electrophoresis on a 2 % agarose gel and then visualized under an Gel Imaging System (Pei Qing, Shang hai, China).

### Polymerase Chain Reaction (PCR)

To determine the limit of the conventional PCR, PCR amplification was carried out with the outer primers (F3s and B3s) for detection of the four *Vibrio* species. The reaction mixture (25 μl) contained 1 μl DNA template, which estimated using the DNA from a tenfold serial dilution of each initial adjusted concentration of bacteria (8 × 10^9^ CFU ml^−1^), 2 μl dNTPs (2.5 mM), 0.1 μl Taq DNA Polymerase with its corresponding buffer, and 0.25 μl each of 20 μM primers. The program comprised 94 °C for 5 min; 25 cycles of 94 °C for 30 s, 55 °C for 50 s and 72 °C for 40 s; and 72 °C for 10 min. The PCR products were subjected to electrophoresis and then visualized as described above.

### Detection and isolation of Vibrio species from infected fish

*V. scophthalmi*, *V. vulnificus*, *V. parahaemolyticu*s and *V. ichthyoenteri* were cultured in Luria–Bertani (LB) medium (OD_600_ ≈ 0.8) at 28 °C and collected by centrifugation. The cells were resuspended in PBS. *Scophthalmus maximus* (averaging 18.4 ± 1.8 g) were purchased from a local fish farm and acclimatized in the laboratory for 2 weeks before experimental manipulation. Fish were fed daily with commercial dry pellets and maintained at 18 °C in tanks supplied with aerated seawater changed daily. Before the experiment, fish were randomly sampled for the examination of bacterial recovery from their blood, liver, kidney, and spleen by the plate-count method, and no bacteria were detected from any of the examined tissues of the sampled fish. Five groups of *Scophthalmus maximus* were injected by intraperitoneal (i. p.) injection with 5 × 10^6^ CFU *V. scophthalmi*, *V. vulnificus*, *V. parahaemolyticu*s, *V. ichthyoenteri* diluted in 100 μl PBS per fish, respectively, and control fishes were injected i. p. with 100 μl PBS. At 24 h post injection, the fish were euthanized, and tissues such as the blood, kidney, spleen and liver were isolated from the tested fish, homogenized and then boiled with an induction cooker to release the DNA. After the quadruplex LAMP reaction ended with the boiled homogenate of the tissue fluid being used as template, the results were detected visually by adding 1 μl (1:10) SYBR Green I into the mixtures, and the color of the positive amplification products changed from orange to green.

Sensitivity of the quadruplex LAMP in infected fish tissues was determined according to method reported by Yu et al. (2013). Briefly, the LAMP reaction was performed using tenfold serial dilutions of the tissue sample prepared above as LAMP templates. The products were subjected to gel electrophoresis. The bacteria number of the detection limits was determined by the following procedure. The pre-boiled diluted homogenates corresponding to the detection limits were plated in triplicate on LB agar plates. The plates were incubated at 28 °C for 48 h, and the colonies that emerged on the plates were counted. The detection limits were also confirmed by visual inspection with fluorescent staining.
